# Patient Management with Metallic Valve Prosthesis during Pregnancy and Postpartum Period

**DOI:** 10.5935/abc.20150130

**Published:** 2015-10

**Authors:** Juliane Dantas Seabra Garcez, Vitor Emer Egypto Rosa, Antonio Sergio de Santis Andrade Lopes, Tarso Augusto Duenhas Accorsi, João Ricardo Cordeiro Fernandes, Pablo Maria Pomerantzeff, Walkiria Samuel Avila, Flavio Tarasoutchi

**Affiliations:** Instituto do Coração – Hospital das Clínicas – Faculdade de Medicina – Universidade de São Paulo – USP, São Paulo, SP – Brazil

**Keywords:** Heart Valve Prosthesis, Thrombosis, Anticoagulants / therapeutic use, Pregnancy, Mortality, Abortion, Spontaneous

## Abstract

Prosthetic thrombosis is a rare complication, but it has high mortality and morbidity. Young women of childbearing age that have prosthetic heart valves are at increased risk of thrombosis during pregnancy due to changes in coagulation factors. Anticoagulation with adequate control and frequent follow-up if pregnancy occurs must be performed in order to prevent complications related to anticoagulant use. Surgery remains the treatment of choice for prosthetic heart valve thrombosis in most clinical conditions.

Patients with metallic prosthetic valves have an estimated 5% risk of thrombosis during pregnancy and maternal mortality of 1.5% related to the event. Anticoagulation with vitamin K antagonists during pregnancy is related to varying degrees of complications at each stage of the pregnancy and postpartum periods. Warfarin sodium crosses the placental barrier and when used in the first trimester of pregnancy is a teratogenic agent, causing 1-3% of malformations characterized by fetal warfarin syndrome and also constitutes a major cause of miscarriage in 10-30% of cases. In the third trimester and at delivery, the use of warfarin is associated with maternal and neonatal bleeding in approximately 5 to 15% of cases, respectively. On the other hand, inadequate anticoagulation, including the suspension of the oral anticoagulants aiming at fetal protection, carries a maternal risk of about 25% of metallic prosthesis thrombosis, particularly in the mitral valve. This fact is also due to the state of maternal hypercoagulability with activation of coagulation factors V, VI, VII, IX, X, platelet activity and fibrinogen synthesis, and decrease in protein S levels.

The Registry of Pregnancy and Cardiac Disease (ROPAC), assessing 212 pregnant women with metal prosthesis, showed that prosthesis thrombosis occurred in 10 (4.7%) patients and maternal hemorrhage in 23.1%, concluding that only 58% of patients with metallic prosthesis had a complication-free pregnancy^1-7^.

There are controversies about the best anticoagulation regimen during pregnancy, childbirth and postpartum of women with metallic valve prosthesis. There are no guidelines about the best single or combined treatment option considering the presumed risk of thrombosis, because there is no evidence regarding maternal effectiveness while taking fetal protection into account. Current recommendations, based on the literature, have been the replacement of warfarin sodium in the first trimester of pregnancy by low-molecular weight heparin (LMWH) until the 12^th^ week of pregnancy. After this gestational age, warfarin is reintroduced until the 36^th^ week of gestation and then replaced again by LMWH 24 hours before delivery^8^. The target INR (International Normalized Ratio) during pregnancy should be 2.5 to 3.5 (mean 3.0) when it is mitral prosthesis, and 2.0 to 3.0 when it is aortic prosthesis, values that give the highest maternal protection rates (5.7% risk of death or thromboembolism) compared with heparine^8^. Published review of pregnant women with prosthetic outcomes showed that warfarin provides better protection than heparin as prophylaxis of thromboembolic events in women with metal prostheses, but with greater risk of embryopathy^9^. However, a retrospective, observational study with 3 anticoagulation regimens: enoxaparin before 6 weeks of pregnancy, between 6‑12 weeks or oral anticoagulants throughout the pregnancy, showed that with the use of enoxaparin, thromboembolic complications were seen in 14.9% and most of them were related to subtherapeutic doses, verified through the measurement of anti-factor Xa^10^. The anticoagulation regimen at subtherapeutic levels is the main cause of valve thrombosis, being found in up to 93% of cases, regardless of the regimen used^11,12^. The risk of thrombosis is probably lower if the anticoagulant dose is appropriate and varies according to the type and position of the metal valve, also taking into consideration the patient's risk factors.

Data from the literature^1,8,9^, warn about the inefficiency of using subcutaneous unfractionated heparin (UFH) in preventing metal prosthetic valve thrombosis during pregnancy, due to difficulties in attaining effective anticoagulation, its control and patient adherence to the drug. However, in services that choose this alternative, it is recommended that UFH be initiated at high doses (17,500-20,000 IU 2xday/subcutaneously) and controlled by activated partial prothrombin time (aPTT), which should be twice the control value, remembering that response to heparin is modified by the physiological state of maternal hypercoagulability. When the LMWH is selected, the dose should be administered every 12 hours, subcutaneously, based on the control of the anti-factor Xa between 0.8‑1.2 U/ml, which should be determined after 4-6h of use. Factors that should be taken into account in deciding the best anticoagulant therapy include: patient preferences, expertise of the attending physician and availability of medication level monitoring^11-14^ (Table 1).

**Table 1:** Anticoagulation in pregnant patient

Time	Medication	Control
Up to 6-12th week	LMWH 1.0 mg/kg SC 12/12h UFH 17.500 to 20.000 IU SC 2x/day	Anti-factor Xa: 0.8-1.2 U/mL aPTT 2x higher than control
12th up to 36th week	Warfarin 5 mg 1x/day orally LMWH 1.0 mg/kg SC 12/12h	INR between 2.0 and 3.0 if aortic prosthesis and between 2.5 and 3.5 if mitral valve prosthesis Anti-factor Xa: 0.8-1.2 U/mL
After 36th week up to delivery	LMWH 1.0 mg/kg SC 12/12h UFH 17,500 to 20,000 IU SC 2x/day	Anti-factor Xa: 0.8-1.2 U/mL aPTT 2x higher than control
Puerperium	LMWH 1.0 mg/kg SC 12/12h Reach target INR after introduction of warfarin 5 mg 1x/day orally	Anti-factor Xa: 0.8-1.2 U/mL INR between 2.0 and 3.0 if aortic prosthesis and between 2.5 and 3.5 if mitral valve prosthesis

LMWH: Low molecular weight heparin; SC: Subcutaneous; UFH: Unfractionated heparin; IU: International units. INR: International normalized ratio.

The European Society of Cardiology contraindicates the use of ASA in addition to anticoagulation in patients with prosthetic valves, as there is no data in the literature demonstrating its benefit and safety^13^. On the other hand, the latest guideline of American Heart Association/American College of Cardiology suggests adding 75-110 mg/day of ASA to the anticoagulation regimen to all patients with metal valves and in patients with biological valves, anticoagulation should be prescribed in the first 3 months and after that maintain ASA at a dose of 75-100 mg/day indefinitely. The addition of aspirin reduces the incidence of embolic phenomena, cardiovascular death and stroke and the Brazilian Society of Cardiology suggests its association in patients with high thromboembolic risk (old prosthesis model in the mitral position, atrial fibrillation, more than one metal prosthesis)^6,15^.

The use of new anticoagulants (direct thrombin inhibitors and Factor Xa oral inhibitors) is formally contraindicated in patients with metallic prosthetic valve.

In the postpartum period, LMWH should be used with Anti‑factor Xa control and subsequent interruption after reaching 3.0 INR with warfarin. During this period, valve thrombosis should be suspected when patients develop progressive dyspnea, pulmonary edema, syncope, symptoms of low cardiac output or hemodynamic instability, after excluding tachyarrhythmias as the cause, especially in patients with inadequate anticoagulation. Additionally, an auscultatory finding that suggests valve thrombosis is the cessation or muffling of the clicking sound when the prosthesis closes. Transesophageal echocardiography seems to be the most sensitive method to confirm the diagnosis^16^.

The treatment of thrombosis during the puerperal period should be the one proposed for patients with prosthetic valve out of the pregnancy and postpartum period, taking into consideration their clinical condition, thrombus size and location of the affected prosthesis. Surgery is the treatment of choice and should preferably be indicated in patients with NYHA functional class III and IV dyspnea, with no surgical contraindication, left prosthesis thrombosis, thrombus ≥ 10 mm or thrombus area > 0.8 cm^2^^6,17^. The disadvantage of surgery is due to high perioperative mortality (between 5%-18%) closely associated with functional class, which is the main predictor. Patients in functional classes I to III (NYHA) have 4-7% mortality, while those in FC IV have 17.5% and 31.3%. However, compared to thrombolysis, surgery has the highest success rates (81% vs. 70.9%)^18,19^.

The use of thrombolytic should be considered in: critical patients at high risk of death if submitted to surgery in places where there is no surgical team available or tricuspid or pulmonary valve thrombosis^20^. Thrombolysis has a systemic embolization risk of 5-19 %, major bleeding 5-8%, recurrence 15-31% and mortality from 6 to 12.5%. Success rates vary from 64 to 89%, with a high chance of being effective if the thrombus has presumably existed for less than 14 days^12,19,21,22^. In case of partial success, or residual thrombus, the patient should be referred to surgery after 24 hours of thrombolytic infusion withdrawal. In this scenario, surgery should be considered an urgency or emergency case, depending on the patient's clinical condition, with high mortality rates. This reinforces the importance of choosing the initial therapy for patients with valve thrombosis, to minimize risks of re-interventions and increase the full resolution rate^19^. Patient monitoring with transesophageal echocardiography should be performed during the procedure. The recommended doses of thrombolytic agents are: streptokinase 1,500,000 IU in 60 min without UFH and Alteplase (rtPA) 10mg in bolus + 90 mg in 90 minutes with UFH^20^. Recently, a thrombolytic protocol with low-dose and slow infusion (rtPA 25 mg intravenous infusion in 6 hours, repeating at 24 h and, if necessary, up to 6x reaching the maximum dose of 150 mg, without bolus or use of concomitant heparin) in pregnant women with prosthetic thrombosis, showed effective thrombolysis with no maternal deaths and fetal mortality around 20%, a better result than the commonly used strategies^11^. However, the author compares it with old studies, and perhaps this difference could be less with the improvement in surgical techniques. Therefore, we can not infer that thrombolysis is better than the surgical strategy in pregnant women.

After surgery or thrombolysis, the patients should be anticoagulated. The US Guidelines advises INR: 3-4 for prostheses in the aortic position and INR: 3.5-4.5 with the addition of aspirin in the mitral position. On the other hand, the European guideline recommends anticoagulation according to the prosthesis thrombogenicity and risk factors for thromboembolic events of the patient (mitral or tricuspid valve, previous thromboembolism, atrial fibrillation, mitral stenosis, ventricular dysfunction EF < 35%), with INR ranging from: 2.5 - 3.5 for low-risk, INR: 3.0 - 4.0 for high risk regardless of prosthesis position^6,20^.

Considering the abovementioned facts, we highlight the importance of warning women of childbearing age that have prosthetic heart valves of the risks during pregnancy, establishing anticoagulation with adequate control and frequent monitoring if pregnancy occurs, preferably at centers of excellence in valvular heart disease, in order to prevent complications related to the use of anticoagulants such as embryopathies, miscarriage, bleeding and prosthesis thrombosis^23^. The treatment should be individualized depending on the patient's clinical condition, according to the our algorithm proposed by our team (Figure 1).

**Figure 1:**
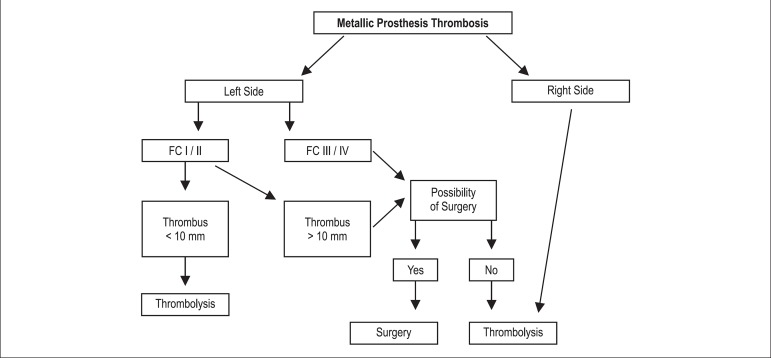
Algorithm proposed for the treatment of prosthetic heart valve thrombosis in pregnant and postpartum women. FC: Functional class the New York Heart Association (NYHA)
